# Retinal Displacement: Providing New Insights for Retinal Detachment Surgery

**DOI:** 10.1155/2021/9999797

**Published:** 2021-08-26

**Authors:** Olivier Houle, Eunice You, Mélanie Hébert, Shicheng Jin, Susan Ruyu Qi, Ali Dirani

**Affiliations:** ^1^Faculté de Médecine, Université de Sherbrooke, Sherbrooke, Canada; ^2^Department of Ophthalmology, CHU de Québec—Université Laval, Québec, Canada; ^3^Department of Ophthalmology, McGill University, Montreal, Canada

## Abstract

**Purpose:**

To review the current literature on retinal displacement and provide a discussion of potential risk factors, postoperative outcomes, and future directions.

**Methods:**

Two databases, MEDLINE and EMBASE, were mined using a directed search strategy to identify all articles on retinal displacement.

**Results:**

We identified 1522 articles. A total of *n* = 14 articles were retained. We provide an overview on the potential influence of surgical type (*n* = 4), tamponade agents (*n* = 5), postoperative posture (*n* = 6), and preoperative retinal status (*n* = 5) on incidence of retinal displacement and visual outcomes (*n* = 8). *Discussion*. Pars plana vitrectomy (PPV) with gas tamponade is associated with displacement rates of up to 72%, typically in a downward direction. Meanwhile, pneumatic retinopexy and PPV with silicone oil may offer similar surgical success with a significantly lower risk of displacement. The impact of heavy liquids such as perfluorocarbon liquid, postoperative positioning and preoperative extent of detachment on displacement remains inconclusive. Patients with displacement had a significantly lower visual acuity and higher rates of distortion than those without displacement. However, not all patients with displacement experienced visual symptoms.

**Conclusion:**

Retinal displacement is a new concept in our understanding of retinal detachment. Additional studies are needed to better define its impact on postsurgical outcomes.

## 1. Introduction

Rhegmatogenous retinal detachment (RRD) is the most common type of retinal detachment, with an annual incidence rate of 6.9–18.2 per 100,000 persons [1]. RRD is primarily treated with either scleral buckling (SB), pars plana vitrectomy (PPV), pneumatic retinopexy, or combined PPV-SB. However, despite successful reattachment and excellent visual acuity (VA) in most patients, up to a third of patients complain of visual distortions postoperatively [2].

In recent years, a concept known as “retinal displacement” has started emerging in the literature. In 2010, Shiragami et al. first reported the previously undescribed morphologic phenomenon occurring after RRD repair [3]. They observed unintentional retinal displacement in up to 62.8% of cases undergoing standard PPV with gas tamponade. This was demonstrated with fundus autofluorescence (FAF) revealing hyperfluorescent lines superior and parallel to retinal vessels. These hyperfluorescent lines, also known as “retinal vessel printings” or alternatively “retinal pigmented epithelium (RPE) ghost vessels” may reflect the augmented metabolic activity of previously hidden RPE now exposed to light following unintentional displacement of retinal vessels postoperatively [4, 5].

A few studies have since suggested that retinal displacement could be responsible for symptoms such as vertical diplopia and distorted vision, which leave patients unsatisfied despite surgical success [4–9]. This study aims to review the literature on potential factors influencing retinal displacement as well as to provide a discussion on postoperative clinical outcomes and future research directions.

## 2. Methods

Two comprehensive databases, MEDLINE and EMBASES, were mined with a directed search strategy using keywords related to retinal or macular displacement (see Supplemental Digital Content 1—Search Strategy). Deduplication was performed through the Endnote software version 8.2 (Clarivate Analytics 2018). The Web-based application Rayyan (https://rayyan.qcri.org) was used to expedite the initial screening of abstracts and titles. References of the eligible studies and relevant review articles were crosschecked to identify additional pertinent studies. No language restrictions were used. We restricted articles to 2010 and onwards.

Studies were included if they evaluated the incidence of retinal displacement after retinal detachment surgery. There were no restrictions on study design, types of retinal detachment surgery, age, comorbidities, or previous medical histories. We excluded studies in which the cause of displacement was not retinal detachment (e.g., epiretinal membrane and macular hole closure), articles that were nonhuman or in vitro studies, articles without an available full-text, and nonrelevant articles. Our main outcome of interest was defined as the presence of retinal displacement as characterized by FAF.

The search strategy was developed by EY and peer-reviewed by senior author AD. The initial title-abstract screening was performed by EY in Nov. 2020 and completeness of search was validated by reviewing the references of recent studies. Afterwards, full texts were retrieved, and two independent reviewers (EY and OH) appraised the papers for eligibility.

Data extraction was achieved by means of a pro forma. The following data were extracted for study characteristics: title of study, first author, year of publication, type of study, total number of patients, female gender, quadrants of retinal detachment, macula status, surgery, use of heavy fluids, and use of tamponade agents. The following data was extracted for outcomes of interest: final surgical outcome (successful vs. failed reattachment), presence of retinal displacement, amount and direction of retinal displacement, visual outcomes, presence of visual symptoms, OCT outcomes, and final follow-up time. Both authors (EY, OH) independently extracted the data. Any disagreements were resolved by open discussion.

## 3. Results

The database search identified 447 articles from Medline and 1132 articles from EMBASE, for a total of 1522 articles when duplicates were excluded by the Endnote software. Initial title-abstract record screening excluded an additional 1479 articles, leaving 27 articles which were retrieved for full-text screening. Citations were crosschecked from existing studies and senior author (AD) was consulted to ensure that key references were not missing. Ultimately, 14 articles were included in the review (see Supplemental Digital Content 2–PRISMA Flow Chart).

All of the included studies employed a prospective design, except for two [6, 7]. In all studies, there were more men than women included. All studies included macula-off patients and 8 studies included both macula-off and macula-on patients [3–5, 7, 10–12]. All studies included PPV patients, Pandya et al. included a single patient with SB [6], Lee et al. included 9 patients treated with SB [5], and Brosh et al. compared PR with PPV [7]. Gas was used as a tamponade agent in all studies, with SO used in 4 studies [5, 7, 10, 11]. Ten out of 14 studies employed a form of postoperative face-down positioning. Shawkat et al. compared a log-roll face-down positioning against a supine positioning [8], Casswell et al. compared the effect of face-down positioning versus support-the-break positioning [13], and Shiragami et al. [12] compared face-down positioning employed immediately and 10 minutes after the end of surgery. Two studies sought to further assess retinal displacement characteristics on imaging modality [5, 14]. Of note, Schawkat et al. 2019 and Guber et al. 2019 use the same cohort to investigate the impact of PFCL and postoperative positioning, respectively, and therefore are referred to as Schawkat/Guber et al. when the same information is presented in both articles [8, 9].

Retinal reattachment was successful in all cases except for one patient in the retrospective case series by Pandya et al. [6]. Occurrence of retinal displacement ranged from 7% in PR [7] to 72% in PPV [5], except for Pandya et al. which was a retrospective case series of 5 retinal displacements. Amount and direction of retinal displacement and presence of visual symptoms were reported in most studies. Six studies assessed patients with OCT postoperatively [4–7, 10, 15]. Significant improvement of visual acuity following surgical repair was observed in all patients in which this outcome was reported. Follow-up time ranged between less than one month and a year. All study characteristics are presented in Table 1 and all outcomes of interest are shown in Table 2.

### 3.1. Surgery Type and Displacement

In the initial study by Shiragami et al., FAF was performed in 43 consecutive patients that underwent standard PPV with sulfur hexafluoride (SF_6_). Superior hyperautofluorescent lines were detected in 27 eyes (62.8%), suggesting a downward displacement of the retina and its major vessels [3]. The majority of studies noted a downward displacement of the retina [3, 6, 7, 9–12, 15]. Only 3 studies reported incidences of upward displacement, associated with the use of silicone oil in PPV in two studies [10, 11] and PR in one study [7]. All subsequent studies, with the exception of three [5–7], assessed retinal displacement solely in PPV patients. The incidence of displacement in PPV ranged between 35% and 72% [3, 5–8, 10, 11, 13, 15, 16]. Pandya et al. included a case of SB in their retrospective case series of five RRD repairs. Displacement was observed in all four PPV cases and the single case of SB despite subretinal fluid drainage via posterior retinotomies [6]. Lee et al. included 9 cases of RRD treated with SB. None showed evidence of macular displacement following surgery compared to 29 of 51 patients treated with PPV [5]. Brosh et al. retrospectively compared retinal displacement between PR and PPV. Displacement was observed in only 7% of PR cases compared to 44.4% of PPV cases (*p* < 0.001) [7].

### 3.2. Tamponading Agent and Displacement

Gas was used as a tamponade agent in all studies, with SO used in 4 studies [5, 7, 10, 11]. In Codenotti et al., 27 patients underwent PPV with either gas (C_3_F_8_) or silicone oil (SO) tamponade. Occurrence of displacement by FAF was significantly associated with type of tamponade (*p*=0.036), with displacement occurring in 10 of 14 eyes (71.4%) that received gas compared to 2 of 9 eyes (22.2%) that received SO [11]. In Dell'Omo et al. 97 of 125 eyes (77.6%) were tamponaded with SF_6_ and 28 eyes (22.4%) with SO. Type of tamponade was the only significant predictor of displacement (*p*=0.007), with displacement occurring in 40 of 97 (41.2%) eyes tamponaded with SF_6_ compared to only 4 of 28 (14.3%) eyes tamponaded with SO [10]. In Lee et al. and Brosh et al., only 2 and 4 cases used SO, respectively; thus no conclusion could be made [5, 7].

Although not included in the initial analysis of the literature, a recently published study by Filippelli et al. also supports a lower incidence of retinal displacement with SO as a tamponade agent. Upward retinal displacement occurred in only 2 out of 44 patients (4.5%) treated with PPV and SO [17].

Meanwhile, the use of adjunctive heavy liquids such as perfluorocarbon liquid (PFCL) is up for debate. In Dell'Omo et al., PFCL did not play a significant role in retinal displacement [10]. In contrast, Schawkat/Guber et al. concluded that displacement occurred less often with patients that were treated with heavy liquids (*p*=0.049), possibly by leaving a smaller amount of subretinal fluid behind [8, 9]. In Brosh et al., only 9 patients of 238 were treated with PFCL and therefore no conclusion was possible [7].

### 3.3. Postoperative Posture and Displacement

In a feasibility study by Dell'Omo et al., strict prone posture was maintained and well tolerated for 2 hours following PPV in 20 eyes. Displacement was observed on FAF in 7 out of 20 eyes (35%), corresponding to a lower rate compared to the literature [16]. In contrast, immediate face-down position with no sitting position for the first 24 hours did not reduce retinal displacement in Codenotti et al., with displacement occurring in most cases [11]. Similarly, the incidence rate of displacement (60%) was not reduced despite face-down positioning following surgery in Cobos et al. [15].

Schawkat et al. performed a randomized controlled trial evaluating a “log-roll” posture, defined as 30 minutes face-to-temporal followed by 30 minutes face-down, against a supine posture for 6 hours, with no significant difference in the two postures [8]. Another study by Casswell et al. compared the effect of face-down positioning vs. a “support-the-break” positioning. Displacement was detected in 42 of 100 eyes (42%) in the face-down group and 58 of 103 eyes (56%) in the “support-the-break” group (*p*=0.04) [13]. Lastly, Shiragami et al. compared face-down positioning initiated 10 minutes after surgery against immediate face-down positioning. Retinal displacement occurred in 28 out of 44 (63.6%) patients in the group with a 10-minute delay compared to only 10 out of 42 (24%) patients in the immediate group (*p*=0.029) [12].

### 3.4. Preoperative Retinal Status and Displacement

In Shiragami et al., extent of retinal detachment (*p*=0.019) and macular status (*p*=0.016) were significantly associated with displacement [3]. In Codenotti et al., displacement occurred in 5 of the 13 macula-on eyes (38.46%) and in 7 of 10 macula-off eyes (70%). These suggest a trend between the number of quadrants involved by RRD preoperatively and the occurrence of retinal displacement postoperatively [11].

In contrast, Schawkat/Guber et al. [8, 9], Dell'Omo et al. [10], and Cobos et al. [15] found no association between preoperative extent of detachment and risk of displacement. Casswell et al. reported no association between the number of quadrants involved, location or number of breaks, and occurrence of retinal displacement [13]. Dell'Omo et al. found no correlation between the direction of retinal displacement and the initial localization of RRD [10]. Studies did not evaluate the presence or severity of PVR.

### 3.5. Postoperative Visual Outcomes and Displacement

A significant improvement in VA compared to baseline was seen in all studies. However, in Brosh et al., patients with displacement achieved a significantly lower final VA with a mean postoperative logMAR of 0.57 vs. 0.35 for patients without displacement (*p* < 0.001) [7]. This is consistent with Casswell et al. in which amplitude of retinal displacement was associated with higher D chart distortion scores (*p*=0.008) and worse VA at 6 months (*p* < 0.001) [13]. In contrast, displacement did not appear to significantly impact VA 12 months postoperatively in Dell'Omo et al. (*p*=0.015) [10].

In Shiragami et al., none of the patients with displacement experienced subjective cyclovertical diplopia or slant although almost half of these patients had objective excyclotorsion or vertical deviation on orthoptic inspection [3]. In Panyda et al. [6], one patient complained of metamorphopsia without evidence of macular abnormalities on OCT. Additionally, three patients had vertical diplopia, consistent with an inferior displacement. One patient eventually recovered. In Schawkat/Guber et al., metamorphopsia was subjectively reported by 10 of 17 patients (58.8%) with displacement [8, 9]. Similarly, in Codenotti et al., 2 of 23 retinal displacement patients reported vertical diplopia and vision distortion [11]. In Lee et al., postoperative distortion was reported in 19 out of 23 patients that showed retinal vessel printings on FAF. The number of displaced vessels sampling points was predictive of visual disturbances in the postoperative period [5].

In Brosh et al., objective measurements of metamorphopsia were carried out in 147/238 patients and testing for aniseikonia was done in 142/238 patients. There were significant differences in incidence of vertical metamorphopsia between displaced retinas and nondisplaced retinas (83.3% vs. 55.6%) (*p*=0.005). However, there were no significant differences for horizontal metamorphopsia (66.7% vs. 53.0%; *p*=0.18) or aniseikonia (51.7% vs. 47.8%; *p*=0.70) [7]. This is consistent with the notion that downward displacement of the retina is primarily responsible for the visual distortions.

### 3.6. Imaging Findings

Among the articles included in this review, 8 used digital fundus camera (FC) and 4 used confocal scanning laser ophthalmoscope (cSLO) [4, 11, 15, 16]. Casswell et al. used both methods in their two studies [13, 14]. In one study, they compared cSLO and FC in 70 eyes with macula-off RRD and found similar results between the two methods, with 88.6% of cSLO images deemed gradable and retinal displacement detectable in 52.8% of cases compared to 87.1% and 61.4% of FC images, respectively [14]. In another study, FC was used primarily, with cSLO being used only in cases of nonconsensus [13].

Six studies assessed patients with OCT postoperatively [4–7, 10, 15]. Brosh et al. identified interdigitation zone abnormalities associated with retinal displacement [7]. Lee et al. identified OCT abnormalities in 15 of 32 (47%) macula-off RRD and 4 of 17 (24%) macula-on patients treated with PPV and gas including retinal folds and subretinal fluid among other findings [5]. Dell'Omo et al. also identified outer and inner retinal folds and subfoveal and intraretinal fluid [4]. No characteristic findings were noted on OCT in the other studies [6, 10, 15].

## 4. Discussion

In 2010, Shiragami et al. first identified hyperfluorescent lines on fundus autofluorescence imaging following surgery of retinal detachment. Termed “retinal vessel printings” by Dell'Omo et al. [18] or “RPE ghost vessels” by Lee et al. [5], those lines were thought to represent unintentional retinal displacement after RRD repair. Shiragama et al. hypothesized that these lines were due to the dramatic increase in metabolic activity once RPE cells previously hidden by vessels were suddenly exposed to light [3]. Alternatively, Dell'Omo et al. postulated that the fluorescent spectral properties and fluorophores within concealed RPE cells inherently differed from those normally exposed to light [18]. This may better explain why retinal vessel printings persist for years after surgery.

PPV is the most common surgical intervention for RRD. The risk of displacement ranges between 35% and 72% [3–13, 15, 16]. The presence of subretinal fluid immediately after surgery may influence displacement although posterior retinotomies did not appear to prevent it [6]. In comparison, PR is a surgical approach that appears to be associated with a decreased risk of retinal displacement without compromising surgical success although it was evaluated in only one study [7]. It was hypothesized that PR allows for a more natural reabsorption of the subretinal fluid by the RPE compared to the forced internal drainage used during PPV. With air-fluid exchange in PPV, there is a near 100% gas fill which exerts a large buoyant force on a larger area of a relatively mobile retina. In comparison, the gas bubble in PR is smaller and contacts a smaller area of the retina, causing less displacement of the subretinal fluid and retina [7]. However, conclusions from Brosh et al. regarding PR may be limited due to the recruitment of patients from three institutions with different preferred surgical approaches, with only one of the three sites commonly performing PR. PR procedures that fail were also converted to PPV. Furthermore, the study is limited by the inclusion of only RRD patients with a single retinal break or a group of retinal breaks in the detached retina in the upper quadrants (within 1 clock hour above the 8- and 4-o'clock meridians) [19]. Further prospective studies will be required to address the potential unknown biases in their study.

Tamponade agents provide additional surface tension on the retina to minimize fluid displacement associated with retinal displacement. In PPV, use of gas as a tamponade agent provides higher risk of displacement [3, 5–16]. In contrast, SO was significantly associated with less displacement compared to gas tamponade in two studies [10, 11]. This difference was thought to be due to the physical properties of silicone oil including its high specific gravity, lower surface tension, and much lower buoyancy in comparison to gas. SO subsequently exerts a much lower force on the retina and may favor a more gradual reabsorption, allowing the retina the time to slowly return to its original position [10]. There may also be benefits to the use of heavy liquids such as PFCL which may promote better drying of the retina and prevent retinal slippage. However, current data is inconclusive [7, 8, 10]. PFCL may also be associated with complications including retained PFCL in up to 10% of patients [9].

Identifying the ideal postoperative patient positioning may allow us to reduce retinal displacement by influencing subretinal fluid behavior. Current studies show contradictory results on the benefits of face-down posture [8, 11–13, 15, 16] although the most recent and largest cohort study on the subject appeared to demonstrate significant reduction of retinal displacement with face-down posturing postoperatively [13], with better outcomes in immediate face-down posturing rather than with delayed posturing [12].

A larger initial extent of retinal detachment including involvement of the macula was found to be a risk factor for retinal displacement by some authors [3, 11], although this link was not substantiated by other studies [8, 10, 15].

There was a significant improvement in VA postoperatively in all the included studies, although patients with retinal displacement achieved a significantly lower final VA than those without displacement [7, 13]. Furthermore, not all patients with displacement complained of visual symptoms even if all patients had sufficient VA to perceive distortions. Others may recover optimal visual function, possibly due to a degree of compensatory sensory fusion [3, 6]. The correlation between displacement and VA is not well understood [7, 13]. Furthermore, while all studies assessed for surgical success and VA, not all evaluated systematically assessed for visual symptoms or did so in a standardized manner across the studies. Achieving high-integrity retinal reattachment could improve alignment of photoreceptors and allow for better functional outcomes [7]. However, the fovea can also be stretched during RRD and contribute to postoperative visual distortions regardless of retinal displacement [13].

Lastly, there is also still room to expand on the role of imaging modalities as there is no agreed gold standard for detection of retinal shift. Confocal scanning laser ophthalmoscope (cSLO) and digital fundus camera (FC) are both commonly used for FAF with moderate agreement between the two modalities. Some differences include diminished autofluorescence of RPE ghost vessels on cSLO (which may make it more challenging to differentiate from background) but more prominent choroidal vasculature on FC pictures (which can confound RPE ghost vessels with choroidal vessels) [14]. cSLO may also be less accessible but less technically challenging, does not require additional barrier filters, and may provide better sensitivity for subtle abnormalities in comparison to FC [14].

OCT may also provide additional insights into morphologic and structural changes that occur postoperatively at the external limiting membrane and photoreceptor inner-segment/outer-segments that lead to visual distortions. Abnormalities that have been identified include outer and inner retinal folds, blebs of subretinal or intraretinal fluid, outer-retinal photoreceptor layer defects, epiretinal membrane, full thickness macular fold, cystoid macular edema, and full thickness macular hole [4, 5]. A combination of FAF and OCT imaging for retinal displacement identification is preferred as FAF changes may lag behind ultrastructural changes on OCT and regress over time.

Future research is therefore warranted to address these gaps in knowledge and identify potential risk factors for retinal displacement including preoperative retinal status (e.g., break size, break number, and extent of the detachment) and choice of intraoperative technique and tamponading agents as well as optimal postoperative protocol. Correlating incidence of displacement and other ultrastructural abnormalities with visual symptoms is a critical area for research in retinal displacement.

### 4.1. Limitations

As the subject is recent, MESH terms were not available and terms to describe retinal displacement could vary in the literature. Our literature search strategy could therefore have missed relevant articles although efforts were made to capture all equivalent terms as well as to ensure completeness of the search (e.g., extensive search strategy of two databases, cross-checking references, etc.)

## 5. Conclusion

Retinal displacement is a niche subject with a lot of ongoing research, including several larger studies published in recent years. Future research will be needed to clarify to compare between the various techniques and methods used in retinal detachment surgery and correlating it with the risk of displacement. Systematic assessments of visual distortions and larger cohorts with long-term follow-up will also be necessary to better evaluate the impact of retinal displacement on functional outcomes given studies are extremely scarce. Having a better understanding of this entity could have significant implications on our clinical decision-making and allow us to better address patient-reported outcomes.

## Figures and Tables

**Table 1:** Study characteristics.

Author, year	Study type	Total number of patients	Female gender	FAF imaging modality	Quadrants of RRD affected	Macular status	Surgery type	Use of heavy fluid	Use of tamponade agent	Postoperative posture
Shiragami [3]	Prospective interventional case series	43	17	FC	1–2 eyes	On = 12	PPV	30	Gas = 43	Sitting several minutes immediately post-op with face-down or appropriate positioning for 7 to 10 days post-op
					2–31 eyes	Off = 15^*∗*^				
					3–8 eyes					
					4–2 eyes					

Pandya [6]	Retrospective case series	5	0	FC	N/A	Off = 5	PPV = 4	N/A	Gas = 5	Face-down post-op
							SB = 1			

Brosh [7]	Retrospective consecutive case series	238	94	FC	>2 = 91 eyes	On = 71	PPV = 124	8	Gas = 117	All but 4 patients in the PPV group had immediate face-down positioning
					≤2 = 138 eyes	Off = 167	PR = 114		SO = 4	

Schawkat [8]	Prospective randomized controlled trial	50	14	FC	1–2 eyes	Off = 50	PPV	25	Gas = 50	Log-roll postoperatively with face-down position vs. lying flat on back for 6 hours
					2–23 eyes					
					3–17 eyes					
					4–8 eyes					

Dell'Omo [10]	Prospective observational case series	125	26	FC	2–67 eyes	On = 23	PPV	80	Gas = 97	Strict prone posture for at least 2 hours in the operating room and during the following 24 hours
					3–26 eyes	Off = 122^†^			SO = 28	
					4–32 eye					

Codenotti [11]	Prospective observational case series	23	8	cSLO	1–6 eyes	On = 13	PPV	23	Gas = 14	All patients treated with gas tamponade were immediately positioned face-down
					2–10 eyes	Off = 10			SO = 9	
					3–3 eyes					
					4–23 eyes					
										Sitting position was avoided for the first 24 hours

Cobos [15]	Prospective observational case series	20	N/A	cSLO	1–5 eyes	On = 12	PPV	N/A	Gas = 20	Face-down immediately post-op
					2–9 eyes	Off = 8				
					3–4 eyes					
					4–2 eyes					

Casswell [13]	Prospective randomized controlled trial	239	68	FC + cSLO	N/A	Off = 239	PPV	3	Gas = 239	Face-down or support-the-break for 24 h in each group followed by support-the-break positioning for 6 days

Dell'Omo [16]	Prospective observational case series	20	8	cSLO	2.95 (0.75)^‡^	Off = 20	PPV	0	Gas = 20	Strict prone posture for 2 hours post-op

Guber [9]	Prospective randomized controlled study	50	14	FC	1–2 eyes	Off = 50	PPV	25	Gas = 50	Group 1: Log-roll (*n* = 26)
					2–23 eyes					Group 2: Lying flat on back for at least 6 hrs (*n* = 24)
					3–17 eyes					
					4–8 eyes					

Lee [5]	Prospective observational case series	60	N/A	FC	PPV-gas in fovea-involving patients:	On = 35	PPV = 51	PFCL = 16/32 PPV + GAS fovea-involving RRD	Gas = 49	Face-down for >1 hr in almost all patients
					1–17 eyes	Off = 37^§^	SB = 9		SO = 2	
					2–9 eyes					
					3/4–5 eyes					

Shiragami [12]	Prospective randomized controlled trial	86	33	FC	1–7 eyes	On = 36	PPV	63	Gas	Group 1: Face-down position approximately 10 to 20 minutes after procedure completion
					2–49 eyes	Off = 50				Group 2: Face-down position immediately after surgery
					3–26 eyes					Both groups maintained appropriate position for the next 7–10 days
					4–4 eyes					

Dell'Omo [4]	Prospective observational case series	33	6	cSLO	1–3	On = 11	PPV	15	Gas	N/A
					2–15	Off = 22				
					3–9					
					4–6					

Casswell [14]	Prospective comparative study	70		FC + cSLO		Off = 70	PPV	0	Gas	Patients randomized to face-down (32/70) or ‘‘bubble-to-break” (38/70) for the first 24 h

^*∗*^Status given for the 27 cases of retinal displacement. †All cases were macula-off, but the fovea was attached in 23 of 125 cases (18.4%) on preoperative OCT examination. ^‡^Mean (SD). PFCL was used, but number not mentioned. Data only available in supplementary file for patients with fovea-involving detachment that received PPV + Gas. ^§^On: 26 PPV, 7 SB; Off: 34 PPV, 3 SB. cSLO = Confocal scanning laser ophthalmoscope; FC = fundus camera; PPV = pars plana vitrectomy; PR = pneumatic retinopexy; SO = silicone oil.

**Table 2:** Outcomes of interest.

Author, year	Final surgical outcome	Presence of retinal displacement N (%)	Amount and direction of retinal displacement	Visual acuity	Presence of visual symptoms	OCT outcomes	Final follow-up time
Shiragami [3]	All successful reattachment	27/43 (62.8%)	Downward = 43 eyes	Significant improvement in all patients	No patients complained of cyclovertical diplopia or slant	N/A	6 months
			1–5° extorsion = 16 eyes				
			1–4° vertical deviation = 13 eyes				

Pandya [6]	1 failed reattachment	5/5 (100%)	Downward = 5 eyes	Significant improvement in all patients	Vertical diplopia reported in 3/5 patients and 1/5 patients at 3-month follow-up	Residual subfoveal fluid = 1/5	Mean follow-up: 5.8 months
						Inferior redetachment = 1/5	

Brosh [7]	All successful reattachment	PPV = 55/124 (44.4%)	Upward:	Significant improvement in all patients	Vertical:	Interdigitation zone abnormalities on the 6 mm OCT:	Median follow-up: 3 months
		PR = 8/114 (7%)	PPV = 3 eyes		With displacement:	With displacement:	
			PR = 6 eyes		25/30 (83.3%)	55/57 eyes (96.5%)	
			Downward:		Without displacement:	Without displacement:	
			PPV = 52 eyes		65/117 (55.6%)	135/162 patients (83.3%) eyes	
			PR = 2 eyes		Horizontal:		
			Mean displacement		With displacement:		
			PPV = 0.297 mm		20/30 (66.7%)		
			PR = 0.137 mm		Without displacement:		
					62/117 (53%)^*∗*^		

Schawkat [8]	All successful reattachment	17/50 (34%)	Ranged between 1.0° and 7.5°	Significant improvement in all patients	10/17 of patients with retinal displacement	N/A	6 weeks

Dell'Omo [10]	All successful reattachment	40/125 (35.2%)^†^	Upward = 5 eyes	Significant improvement in all patients	No patients complained of cyclovertical diplopia or slant.	OCT sections of RVPs did not reveal characteristic features	12 months
			Downward = 39 eyes				
			18–58° extorsion = 28 eyes				
			18–38° intorsion = 3 eyes				
			18–48° vertical deviation = 13 eyes				

Codenotti [11]	All successful reattachment	12/23 (52.2%)^‡^	Downward = 10 eyes	Significant improvement in all patients	2/23 complained of vertical diplopia and vision distortion	N/A	3 months
			Upward = 2 eyes				

Cobos [15]	All successful reattachment	12/20 (60%)	Downward = 12 eyes	Significant improvement in all patients	N/A	OCT analysis showed no differences in retinal displacement cases	3 months

Casswell [13]	All successful reattachment	100/203 (49.26%)^*∗∗*^	Face-down = 0.5° at 8 weeks, 0.3° at 6 months	Significant improvement in all patients	More diplopia reported in face-down patients (1.5%) compared to support-the-break (7.6%)	N/A	6 months
			Support-the-break = 0.8° at 8 weeks, 0.9° at 6 months				

Dell'Omo [16]	All successful reattachment	7/20 (35%)	N/A	Significant improvement in all patients	N/A	N/A	4 weeks

Guber [9]	All successful reattachment	17/50 (34%)	Downward shift	Significant improvement in all patients	10/17 with retinal shift reported distorted vision	N/A	2 months
			Shift between 1.0° and 7.5°				

Lee [5]	All successful reattachment	PPV + gas:	Displacement is typically heterogeneous	N/A	PPV + gas:	PPV + gas:	Median follow-up: 29 days
		Mac-off = 22/32 (72%)	Magnitude of displacement associated with extent of retinal detachment in degrees of involvement above (*p*=0.0003) and below the fovea (*p*=0.02)		Mac-off = 22/32 (72%)	Mac-off = 15/32 (47%) ORF (*n* = 6); SRF (*n* = 1); outer-retinal photoreceptor layer defects (*n* = 3); ERM (*n* = 1); full thickness macular fold (*n* = 1) or hole (*n* = 1)	
		Mac-on = 5/17 (29%)			Mac-on = 2/17 (12%)^§^	Mac-on = 4/17 (24%)	
		PPV + SO : Mac-off = 2/2 (100%)				ORF (*n* = 2); CME (*n* = 1); ERM (*n* = 1)	

Shiragami [12]	All successful reattachment	38/86 (44.2%)	Downward shift	N/A	Group 1 = 1/44 patients	N/A	3 months
			1–7° extorsion				
			1–9° vertical deviation				

Dell'Omo [4]	All successful reattachment	4/33 (12.1%)	N/A	Significant improvement in all patients	22 (67%) complained of postoperative metamorphopsia	ORF = 14 (42.4%)	Median follow-up: 24 days
						IRF = 16 (48.8%)	
						IS/OS skip reflectivity abnormalities = 8 (24.2%)	
						Photoreceptor IS/OS band disruption at the fovea = 10 (30.3%)	

Casswell [14]	All successful reattachment	61.4% of FC images	N/A	N/A	N/A	N/A	8 weeks
		52.8% of cSLO images					

^*∗*^147 patients were evaluated for metamorphopsia; ^†^40 eyes in the SF6 group and 4 eyes in the SO group. ^‡^Occurrence of retinal displacement was higher in eyes with gas tamponade (10 of 14 eyes; 71.4%) compared with eyes with silicone oil (2 of 9 eyes; 22.2%) (*p*=0.036). ^*∗∗*^At 6 months, retinal displacement was detected in 42 of 100 (42%) in the face-down positioning group vs. 58 of 103 (56%) in the support-the-break positioning group (odds ratio, 1.77; 95% CI, 1.01–3.11; *p*=0.04). ^§^Of those with retinal shift, symptoms were present in 83% (19/23). Of those with evidence of shift, symptoms were present in 86% (19/22). CME  =  cystoid macular edema; ERM  =  epiretinal membrane; N/A = not available; ORF = outer-retinal fold.

## Abbreviations

FAF: Fundus autofluorescence
FC: Fundus camera
cSLO: Confocal scanning laser ophthalmoscopy
OCT: Optical coherence tomography
PFCL: Perfluorocarbon liquid
PPV: Pars plana vitrectomy
RPE: Retinal pigmented epithelium
RRD: Rhegmatogenous retinal detachment
SB: Scleral buckling
SO: Silicone oil
PR: Pneumatic retinopexy
VA: Visual acuity.

## Additional Points

*Summary*. The authors present the first review on retinal displacement, an exciting concept that may provide important insights on postsurgical outcomes in retinal detachment repair. They provide a complete overview of all existing research to date as well as identifying research gaps.

## Supplementary Materials

Supplementary Materials Appendix 1: search Strategy (Supplemental Digital Content 1.docx); Appendix 2: PRISMA Flow Chart (Supplemental Digital Content 2.docx).
