# Supplementary material for: Retinal Displacement: Providing New Insights for Retinal Detachment Surgery
Source: J Ophthalmol. 2021 Aug 26;2021:9999797. doi: 10.1155/2021/9999797 (PMC8413052; doi:10.1155/2021/9999797)
Supplement: Supplementary Materials — Appendix 1: search Strategy (Supplemental Digital Content 1.docx); Appendix 2: PRISMA Flow Chart (Supplemental Digital Content 2.docx). [file 9999797.f1.docx]

**APPENDIX 1**

**Search strategies**

*MEDLINE via OVID search*

1. retina* displacement.mp.

2. macula* displacement.mp.

3. retina* slippage.mp.

4. macula* slippage.mp.

5. retina* shift.mp.

6. macula* shift.mp.

7. 1 or 2 or 3 or 4 or 5 or 6

8. exp Retinal Detachment/ .

9. exp Vitrectomy/

10. exp Scleral Buckling/

11. retinopexy.mp.

12. retina* reattachment.mp.

13. macula* reattachment.mp.

14. 8 or 9 or 10 or 11 or 12 or 13

15. metamorphopsia.mp.

16. visual distortion.mp.

17. dysmetropsia.mp.

18. autofluorescence.mp.

19. fundus autofluorescence.mp.

20. exp retinal vessels/ or retina* vessel.mp.

21. retinal vessel printing.mp.

22. retina* ghost vessel.mp.

23. 15 or 16 or 17 or 18 or 19 or 20 or 21 or 22

24. 14 and 23

25. 7 or 24

26. limit 25 to yr= “2010-Current”

Total = 447 articles

*EMBASE via OVID search*

1. retina* displacement.mp.

2. macula* displacement.mp.

3. retina* slippage.mp.

4. macula* slippage.mp.

5. retina* shift.mp.

6. macula* shift.mp.

7. 1 or 2 or 3 or 4 or 5 or 6

8. exp retina detachment/

9. exp vitrectomy/ or exp pars plana vitrectomy/

10. exp sclera buckling procedure/ or exp scleral buckle/

11. exp retinopexy/

12. retina* reattachment.mp.

13. macula* reattachment.mp.

14. 8 or 9 or 10 or 11 or 12 or 13

15. exp metamorphopsia/

16. visual distortion.mp.

17. dysmetropsia.mp.

18. exp autofluorescence imaging/ or exp autofluorescence/

19. fundus autofluorescence.mp.

20. exp retina blood vessel/

21. retinal vessel printing.mp. or retina* ghost vessel.mp.

22. 15 or 16 or 17 or 18 or 19 or 20 or 21

23. 14 and 22

24. 7 or 23

25. limit 24 to yr= “2010 -Current”

Total = 1132 articles

**APPENDIX 2**

**PRISMA flowchart**

Records identified through database searching
(n = 1579)

Full-text articles excluded, with reasons (n= 18)

Epiretinal membrane surgery n = 7

Macular hole surgery

n = 4

Internal limiting membrane n = 2

In vitro study n = 1

Foreign language n = 0

Correspondence, supplement, or conference abstract

n = 4

## Included

## Eligibility

## Identification

## Screening

Records excluded
(n =1479)

Records screened
(n = 1522)

Records after duplicates removed
(n = 1522)

Studies included in qualitative synthesis
(n = 9)

Full-text articles assessed for eligibility
(n = 27)

Medline: 447; EMBASE:1132

Additional records identified through other sources
(n = 0)
